# Efficacy of potential chemical control compounds for removing invasive American bullfrogs (*Rana catesbeiana*)

**DOI:** 10.1186/s40064-015-1319-6

**Published:** 2015-09-15

**Authors:** Gary W. Witmer, Nathan P. Snow, Rachael S. Moulton

**Affiliations:** USDA/APHIS Wildlife Services, National Wildlife Research Center, 4101 Laporte Avenue, Fort Collins, CO 80521-2154 USA

**Keywords:** Amphibian, Caffeine, Chloroxylenol, Colorado, Integrated pest management, Permethrin, Rotenone, Toxicant

## Abstract

Invasive American bullfrogs [*Rana catesbeiana* (*Lithobates catesbeianus*)] are outcompeting and predating on native biota and contributing to reductions in biodiversity worldwide. Current methods for controlling American bullfrogs are incapable of stopping their expansion, thus more cost-effective and broadly applicable methods are needed. Although chemical control compounds have been identified as effective for removing other invasive amphibians, none have been tested for American bullfrogs. Our objective was to expand on previous research and test the efficacy of 10 potential chemical control compounds for removing invasive American bullfrogs. After a dermal spray-application of 4 ml, we found 3 compounds (i.e., chloroxylenol, rotenone with permethrin, and caffeine) at 5–10 % concentrations in water were 100 % lethal for adult American bullfrogs. Chloroxylenol and rotenone with permethrin were fast acting with time-to-death <2 h. This research presents a first-step toward incorporating chemical control as part of integrated pest management strategy for controlling invasive American bullfrogs. Follow-up studies on delivery systems and reducing non-target hazards should ensue with these compounds to confirm their effectiveness and safety for removing invasive American bullfrogs.

## Background

The lack of social acceptance and high ecological risk associated with chemical control strategies has limited their widespread use on invasive amphibian species. However, when invasive amphibians are threatening the persistence of native biota and directly contributing to declines in biodiversity, the use of suppression agents may be warranted. In many situations, invasive amphibian species are threatening population persistence of native biota and are major nuisances (Kraus et al. 1999; Lever 2003). However, relatively few management options exist to control these invaders. This lack of options has become exposed again by another emerging invasive amphibian, the American bullfrog [*Rana catesbeiana* (*Lithobates catesbeianus*)], that is devastating aquatic biota worldwide (Adams and Pearl 2007; Snow and Witmer 2010).

From 1940–1990, American bullfrogs were introduced as a food source worldwide and populations now exist throughout western North America, Oceania (Pacific Ocean islands), Asia, Europe, the Caribbean, and South America (Staples and Cowie 2001; Witmer and Lewis 2001; Lever 2003; Govindarajulu 2004; Boersma et al. 2006; Palen 2006). Since their introduction, many populations have expanded their range. American bullfrogs are responsible for negative ecological effects, including declines in native biota from intense predation and competition (Kats and Ferrer 2003; Snow and Witmer 2010). American bullfrogs have been difficult to control because of their high mobility, generalized eating habits, and high reproductive capacity (Moyle 1973; Adams and Pearl 2007). Both tadpoles and adult bullfrogs are voracious feeders and can consume benthic algae and the eggs and young of native invertebrates and vertebrates (Bury and Whelan 1984). Once established, invasive American bullfrogs directly compete with native birds, reptiles, amphibians and fishes for resources (Bury and Whelan 1984; D’Amore 2012). Additionally, the tadpoles of American bullfrogs alter aquatic vegetation by regulating primary production and nutrient cycling (e.g., Pryor 2003).

The challenges of managing American bullfrogs are categorized into 3 main categories, (1) bullfrogs are well established following a broad invasion, (2) their removal has not yet generated much financial support, and (3) there is a scarcity of control methods for them (Adams and Pearl 2007). Current control methods include direct removal such as hand capture, netting, trapping, spearing (gigging), and shooting (Rosen and Schwalbe 1995; Banks et al. 2000; Snow and Witmer 2011; Louette et al. 2013, 2014). These techniques have limited ability to eradicate bullfrogs because they exhibit strong density dependence and experience increased reproduction and survival if not all bullfrogs are removed (Werner et al. 1995; Altwegg 2002; Doubledee et al. 2003; Govindarajulu 2004). These methods are also labor and time intensive. Another control method includes habitat manipulation such as draining ponds (Doubledee et al. 2003; Adams and Pearl 2007), but the efficacy and secondary effects on native species are not well understood (e.g., Maret et al. 2006).

There are currently no chemical control compounds registered for use against invasive American Bullfrogs. Chemical control has proven to be cost effective and successful for reducing other invasive frogs over large areas (e.g., Campbell and Kraus 2002). Rotenone, for example, is used to rid water bodies of unwanted fish and potentially bullfrogs (Finlayson et al. 2000; Rayner and Creese 2006). Carbon dioxide can be used to suppress bullfrog larvae, but not adults (Abbey-Lambertz et al. 2014). If chemical control could be integrated into other management strategies, such as reducing water and then applying a compound (e.g., Rayner and Creese 2006), chemical control may assist in removing bullfrogs. Integrated approaches to the management and control of invaders are the most effective way to stem the spread of invasive species.

A potentially more cost effective and broadly applicable technique, such as chemical control, is needed for integrated pest management strategies to successfully remove invasive American bullfrogs. Our objective was to determine the effectiveness of 10 potential chemical control compounds on the survival of adult American bullfrogs under controlled, laboratory conditions at the USDA National Wildlife Research Center in Fort Collins, Colorado. We limited our testing of compounds to those compounds used in previous work with Coqui frogs (e.g., Pitt et al. 2005), cane toads (e.g., D. J. Dall, Pestat Party Ltd., personal communication), and other aquatic control programs (e.g., Rayner and Creese 2006; Schofield and Nico 2007).

## Methods

We obtained 60 free-ranging American bullfrogs from a commercial supplier (Ward’s Natural Science, Rochester, New York) and by live-capturing from a pond near Pueblo, CO. We recorded the initial weight and sexed each bullfrog by comparing the diameter of the tympanum to the diameter of the eye on each individual (George 1938; Bury and Whelan^1984^). The bullfrogs were held for 10 days to acclimate to a laboratory setting before the trial began. Prior to the study, all bullfrogs were group housed in large tanks containing about 8 cm of water, and fed a maintenance diet of crickets, mealworms, and goldfish.

We mixed solutions of caffeine (Sigma-Aldrich, St. Louis, MO), chloroxylenol (chlorodimethylphenol; Sigma-Aldrich), rotenone and permethrin (Liquid Rotenone-Pyrethrins Spray, Bonide Products Inc., Oriskany, New York), Permethrin (Tengard^®^, United Phosphorus Inc., Trenton, New Jersey), rotenone (Rotenone dust, Bonide Products Inc.), calcium hydroxide (hydrated lime; Sigma-Aldrich), citric acid (Sigma-Aldrich), potassium bicarbonate (Sigma-Aldrich), sodium bicarbonate (Arm and Hammer, Princeton, New Jersey), and sodium benzoate (Sigma-Aldrich). We attempted to use concentration levels for each potential chemical control compound that was effective for other invasive species (Table 1). We used tap water as a control treatment.

**Table 1:** Summary of laboratory trials with potential chemical control compounds for invasive American bullfrogs, using dorsally sprayed solutions

Chemical control compound (concentration)	*n*	Body weight		Deaths	Percent mortality	Time to death (h)	Efficacy compared to control	
		Mean (kg)	SE				*Χ*_*1*_^*2*^	*P*
Chloroxylenol (5 %)	5	0.28	0.01	5	100	<2	10.00	0.008
Rotenone (1 %) and permethrin (4.6 %)	5	0.30	0.02	5	100	<2	10.00	0.008
Caffeine (10 %)	5	0.39	0.03	5	100	<16	10.00	0.008
Rotenone (1 %)	5	0.31	0.02	2	40	<43	2.50	0.444
Permethrin (4.6 %)	5	0.37	0.01	2	40	<72	2.50	0.444
Calcium hydroxide (6 %)	5	0.39	0.02	0	0	NA	NA	NA
Citric acid (16 %)	5	0.39	0.02	0	0	NA	NA	NA
Potassium bicarbonate (18 %)	5	0.29	0.02	0	0	NA	NA	NA
Sodium bicarbonate (15 %)	5	0.42	0.01	0	0	NA	NA	NA
Sodium benzoate (15 %)	5	0.40	0.01	0	0	NA	NA	NA
Control (water)	10	0.33	0.02	0	0	NA	NA	NA

*n* The number of bullfrogs in the treatment group, *NA* not applicable

We randomly assigned 5 bullfrogs to each treatment group, ensuring a mixture of 2–3 males and females per group. Each bullfrog was placed in a ceramic bowl (~15 cm diameter × 8 cm tall) and covered with wire mesh that prevented the bullfrog from escaping, but allowed adequate movement and air supply. The bottom of the bowl contained a piece of filter paper and approximately 3 cm of water to keep the bullfrog hydrated. We applied about 4 ml of each treatment solution by spraying onto the entire dorsal surface of each bullfrog using a hand-held plastic spray bottle. We assumed that 4 ml of spray represented a reasonable dosing that could be applied in a one-spray application in the wild. Once treated, bullfrogs were observed for 15 min, then again at 2 and 4 h post exposure, and then twice daily for the next 2 days. Any changes in condition and mortality were recorded during each observation period. If no mortality occurred in the treatment group by the end of day 2, the bullfrogs were humanely euthanized by pithing the brain and spinal cord followed by the removal of the head with scissors (AVMA 2001).

We compared the average weight of bullfrogs in each treatments group with analysis of variance procedures (Proc GLM, SAS Institute, Cary, NC). Then, we compared the efficacy of each potential chemical control compound to results from the group of control American bullfrogs using Fisher’s Exact, Chi squared tests (Proc Freq). We rejected the null hypothesis that the treatments were equally efficacious to the control compounds for producing mortality in bullfrogs at the level of α = 0.05.

## Results

The average weights of adult bullfrogs in each group were variable from 0.28–0.42 kg (F_58_ = 22.95, *P* < 0.0001; Table 1). We identified chloroxylenol (5 %), rotenone (1 %) with permethrin (4.6 %), and caffeine (10 %) as highly efficacious compounds for bullfrogs, compared to the control. These three compounds produced 100 % mortality. In our 5 treatments that caused some mortality, the chloroxylenol treatment and rotenone with permethrin treatment were the fastest acting (less than 2 h to death) of all the potential chemical control compounds. No other compounds were identified as significantly different from the control treatment. Individually, the rotenone (1 %) treatment and the permethrin (4.6 %) treatments produced some mortality (40 %), but were not highly efficacious at the concentrations tested. The three chemical treatments that caused 100 % mortality were effective with both male and female bullfrogs as each treatment group contained members of both genders.

## Discussion

This represents the first study to successfully identify potential chemical control compounds for controlling invasive adult American bullfrogs. This is an important first-step for producing a more broadly applicable and cost effective strategy for controlling this expanding invasive species. We identified two promising compounds that were highly efficacious and fast acting (chloroxylenol treatment and rotenone with permethrin). Another compound was highly efficacious and slower acting (caffeine), and two other compounds may prove more effective at higher concentrations (rotenone and permethrin). Overall, these findings provide a useful baseline for developing a chemical control compound for registered use on bullfrogs. However, we acknowledge that there are still many questions that need investigation before chemical control of bullfrogs can become a viable option.

The compounds we identified as the most effective for killing bullfrogs are already registered for controlling other pests. Chloroxylenol is a broad spectrum antimicrobial chemical that is known to be toxic to fish and aquatic invertebrates but not to mammals (US Environmental Protection Agency 1994), and is an active ingredient used to control cane toads in Australia (Kelehear et al. 2012). Rotenone is an effective piscicide used in fisheries management to control invasive fish (Smith 1940). Permethrin is a common insecticide that is also toxic to fish but not to mammals (Hill 1989).

A primary drawback of using these compounds as chemical control agents is the chemical must come in direct contact with the frog (Pitt and Sin 2004; Pitt and Doratt 2005; Tuttle et al. 2008). Therefore a spray application must have access to all frogs to be effective. For bodies of water that are heavily infested with bullfrogs, water may need to be drained to a manageable level for a spray to be broadcast. Chemical control of bullfrogs will not be applicable everywhere, but perhaps in the most severe infestations where total rehabilitation of the water body is deemed necessary. Water quality concerns (e.g., The US Federal Clean Water Act) and potential impacts to native aquatic species will restrict the direct application of chemicals to water bodies unless total restoration of the water body is the overall objective. In waters heavily infested with bullfrogs, this will likely be the primary objective because they may harbor source populations of bullfrogs that could spread to other nearby water bodies.

Our results suggest that American bullfrogs are more resistant to dermal chemical control compound sprays than are invasive coqui frogs (Pitt et al. 2005). Coqui frogs spend considerably more time out of water than American bullfrogs. Therefore, American bullfrogs are likely more susceptible to desiccation when out of water and have a lower ability to prevent movement of these chemical compounds across skin membranes. This might reduce the ability for dermal-sprayed materials to cross the membranes and produce their adverse effects on the American bullfrog.

## Conclusions

Bullfrogs are a destructive invasive species where they have been introduced. This work provides the foundation for further evaluation of 5 % chloroxylenol, rotenone (1 %), and permethrin (4.6 %), and caffeine (10 %) as potential suppression agents for American bullfrogs. This research, however, is only a first-step toward incorporating chemical control as part of integrated pest management strategy for controlling invasive American bullfrogs because many other aspects of chemical use will need to be addressed before it becomes a viable option. Follow-up studies on chemical concentration, delivery systems, and reducing non-target hazards should ensue with these compounds to confirm their effectiveness and safety for removing invasive American bullfrogs.
